# Staying in the ‘sweet spot’: A resilience-based analysis of the lived experience of low-risk drinking and abstention among British youth

**DOI:** 10.1080/08870446.2015.1070852

**Published:** 2015-11-16

**Authors:** Rebecca Graber, Richard de Visser, Charles Abraham, Anjum Memon, Angie Hart, Kate Hunt

**Affiliations:** ^a^ School of Psychology, University of Sussex, Falmer, UK; ^b^ University of Exeter Medical School, Exeter, UK; ^c^ Division of Primary Care and Public Health, Brighton and Sussex Medical School, University of Brighton, Falmer, UK; ^d^ Community University Partnership Programme, University of Brighton, Falmer, UK; ^e^ MRC/CSO Social and Public Health Sciences Unit, University of Glasgow, Glasgow, UK

**Keywords:** alcohol, adolescence, resilience, protective mechanisms, peer relationships

## Abstract

***Objective:*** The aim of this study was to understand how and why young people drink less or not at all when with their peers. Understanding the subjective experiences of moderate or non-drinkers may help identify protective processes facilitating resilience to cultural norm and influences that encourage excessive alcohol consumption among young people.

***Design:*** Semi-structured interviews were conducted with 25 moderate- or non-drinkers aged 17–25 years (13 young women) living in South East England. Interviews explored recent experiences of social situations and encounters that did or did not involve alcohol. Transcripts were analysed using Interpretative Phenomenological Analysis.

***Results:*** Analysis identified six conceptually coherent themes clustering within a superordinate theme of a healthy experience of moderate alcohol use or abstention: ‘the sweet spot’. These themes were: feeling good in the body, feeling like you can be who you are, feeling like you belong, making a free choice, enjoying the moment, and feeling safe and secure.

***Conclusions:*** This resilience-based analysis showed how non-drinking and moderate-drinking may be experienced as a positive and proactive choice. Understanding the subjective experiences of young people may aid development of specific, realistic interventions to promote moderate drinking and abstention among young people in drinking cultures.

## Introduction

Encouraging young people to drink less can have compelling benefits for their health and psychological well-being, and for public health. Alcohol consumption is one of the world’s leading risk factors for disease and injury, with the highest proportion of the disease burden carried by people aged 15–29 years (Rehm et al., 2009). Young people face short- and long-term health risks from alcohol use such as early sexual activity, psychological distress and hospitalisation for alcohol-related harm (Balogun, Koyanagi, Stickley, Gilmore, & Shibuya, 2014; Healey, Rahman, Faizal, & Kinderman, 2014; Philips-Howard et al., 2010). Alcohol consumption generally, and heavy episodic drinking (so-called ‘binge drinking’) in particular, in this age group is common in many countries: for example, one UK-based study of secondary school students found that 56% had engaged in binge drinking at least once within the past week (Danielsson, Wennberg, Hibell, & Romelsjö, 2011; Dodd, Al-Nakeeb, Nevill, & Forshaw, 2010). Researchers, practitioners and policymakers therefore have persuasive reasons to seek overall reductions in the amount of alcohol consumed by young people and in the frequency of excessive drinking. In cultures where alcohol use is the norm, those concerned with limiting the disease burden may opt to focus on the adaptive processes enabling ‘low risk’ alcohol use among the majority of young people who are likely to choose to drink. This adaptive approach to behaviour change may complement traditional targeted interventions aimed at the comparatively fewer ‘high risk’ drinkers who regularly drink to excess (Danielsson et al., 2011).

An adaptive approach may be particularly suitable in the ‘drinking cultures’ common to young people’s social experience within the UK and internationally (de Visser, Wheeler, Abraham, & Smith, 2013; Kloep, Hendry, Ingebrigtsen, Glendinning, & Espnes, 2001; Roberts, Townshend, Pappalepore, & Eldridge, 2012). Young people’s drinking behaviour is influenced by motivations towards camaraderie, peer acceptance, and beliefs that alcohol can facilitate social interactions (Kuntsche, Knibbe, Gmel, & Engels, 2005; Kuntsche, Rehm, & Gmel, 2004). Although abstinence from alcohol is a medically desirable behaviour, it may not be feasible or realistic in cultures where alcohol has powerful social significance for young people (Frederiksen, Bakke, & Dalum, 2012).

Health-promoting alcohol interventions may benefit from knowledge about comparatively *healthy* drinking behaviour using *adaptive* processes. Understanding ‘best practice’ in alcohol consumption addresses a need to develop pro-active messages about how to limit drinking, with reassurances that this is possible to do (Abraham, Southby, Quandte, Krahé, & van der Sluijs, 2007). Of course in doing so, we run into an immediate paradox: there is, arguably, no such thing as ‘good drinking’ given the public health burden of alcohol consumption and the cumulative risk of excessive drinking for individuals. The UK Chief Medical Officer advises that an alcohol-free childhood is the healthiest option (Department of Health, 2009). However, alcohol is embedded within the cultural landscape: every young person must choose whether, and how much, to drink. Engaging with young people’s subjective experience of their social worlds is therefore essential. Despite this, public health campaigns tend to avoid the social nature of much alcohol use, and are consequently often perceived as irrelevant (de Visser et al., 2013). Interventions focusing on relatively healthy drinking behaviours may provide young people with a realistic goal that can be achieved within their cultural context. Understanding the lived experience of young people who have generally healthy alcohol consumption can therefore provide a psychological operationalisation of low-risk drinking with utility for researchers, parents and practitioners where abstention is unlikely to be a popular choice.

A resilience approach is a useful analytical frame for examining healthy behaviour patterns and adaptive processes. Resilience is a multi-dimensional process whereby young people show positive adaptation in the face of risk or adversity. It incorporates domain-specific and generalised interactive protective mechanisms at the individual level (e.g. effective coping skills, social support and self-efficacy) and the level of social and cultural context (Hart, Blincow, & Thomas, 2007; Luthar, Cicchetti, & Becker, 2000; Rutter, 1990). In a drinking culture where each person is, to some extent, exposed to alcohol-related risk, one observable indicator of resilience may be falling below the threshold for high-risk alcohol consumption. It is, however, unclear what the subjective experience of this resilience may be, or what constellation of generalised or domain-specific features might constitute its realisation.

A resilience-based analysis of healthy behaviour patterns in alcohol use therefore extends the literature in four ways. First, we may frame ‘resilience’ in this context as abstaining or maintaining low-risk drinking patterns in a culture where drinking and heavy drinking are common. Second, the approach sensitizes researchers to adaptive processes that are conceptually distinct from processes that promote risk (Rutter, 1990). Previous research has been understandably focused on unhealthy behaviour patterns such as drinking to cope (e.g. Bernstein, Graczyk, Lawrence, Bernstein, & Strunin, 2011; Nolen-Hoeksema, Wisco, & Lyubomirsky, 2008). A resilience-based analysis may help identify protective mechanisms facilitating adaptive experiences. Third, this approach encourages research with people demonstrating positive adaptation, as opposed to those with maladaptive behaviour patterns. There is comparatively little research into how and why some young people drink little or not at all. Qualitative researchers are beginning to attend to these individuals’ practices, discourses and experiences (Conroy & de Visser, 2013; Frederiksen et al., 2012; Herring, Bayley, & Hurcombe, 2013). Shifting focus can lead to very different interpretations to those offered by dominant cultural paradigms: for example, reframing alcohol abstention or restraint into proactive choices characterised by pride, determination and social integration (Herring et al., 2013). Finally, a resilience approach offers benefits for intervention development by valuing knowledge about positive choices, developing strengths, and facilitating social skills to promote responsible alcohol use and abstinence, whereas a traditional approach focuses on risk avoidance.

The aim of this study was to develop understanding of the experiences of young people within a culture where drinking is common in order to operationalise psychologically healthy alcohol-related behaviour. A resilience-based approach focused on how young people engaged with personal and social encounters in ways they believed to be adaptive, and which aligned with Government guidelines for low-risk alcohol use (Department of Health, 2013).

## Method

### Recruitment and participants

Participants were invited to take part in an interview as part of a large mixed-method study of experiences of alcohol use and non-use among young people aged 16–21 years living in South-East England (de Visser et al., 2014). In line with a resilience-based approach, we specifically sought non-drinkers (including former drinkers) or moderate drinkers, with the rationale that this status was indicative of adaptive behaviour patterns. Status was determined by participants’ scores on the 10-item Alcohol Use Disorders Identification Test (AUDIT; Babor, Higgins-Biddle, Saunders, & Monteiro, 2001) and by two alcohol use history questions identifying whether participants had ever consumed alcohol and if they had consumed alcohol in the last year. We purposively sampled across gender, alcohol use (moderate-drinkers and non-drinkers) and age (either above or below the legal alcohol purchase age of 18 years). An age range inclusive of participants who were either above or below the legal purchase age enabled analysis of illegal and legal drinking, different drinking contexts, and changes over time. Recruitment continued until we obtained at least four young men and young women in the older (18–25 years) and younger (16–17 years) age groups, which is an acceptable sample size for an IPA study (Smith, Flowers, & Larkin, 2009). We also recruited through universities and community-based youth organisations.

Twenty-five young people (13 women) aged 17–25 years participated: 9 younger moderate drinkers, 8 older moderate drinkers, 7 older non-drinkers, and 1 younger non-drinker. Nineteen participants were of ‘white British’ or ‘other white’ background; four were South Asian; one was of mixed White and South Asian descent; and one was Chinese. Most, but not all, participants were students. Fifteen were in full-time education. Seven worked alongside full-time study. One was solely in part-time employment, one was employed full-time, and one was not employed or in education.

### Data collection and generation

Ethical approval was granted by the host university. Semi-structured interviews lasting between 40 and 110 min were conducted by the first author, a 28-year old White woman: 23 in a private room at the host university, one at a college and one at a community organisation. Interviews focused on participants’ experiences of social situations in which they had the option of consuming alcohol to provide a rich account of the lifeworld suitable for interpretative phenomenological analysis (Smith et al., 2009). Questions focused primarily upon experiences of the past 12 months. Participants were asked how their choices related to their friendships; how they found being a non-drinker/moderate drinker; reasons for their drinking choices; and their advice for other young people. Each participant was given a £7 voucher and provided with contact information for the researchers and support organisations. Audio recordings were transcribed. Identifying information was replaced with pseudonyms.

### Analytical procedure

Analysis aimed to understand how young people experience their choices and practices regarding non- and moderate-drinking in a peer context. Interpretative Phenomenological Analysis (Smith et al., 2009) was selected for its methodological emphasis on understanding how individuals sharing a common phenomenon make meaning from their experiences (in this case, of low-risk drinking and abstention). In keeping with a resilience-based analysis, we sought to focus on how participants interpreted their experiences to be subjectively adaptive for the self, their health and their relationships. Following initial immersive readings, each transcript was carefully coded to detail participants’ subjective experiences concerning their alcohol use, including thoughts, behaviours and feelings. Developing patterns were then explored across and within accounts. Commonalities and differences were interrogated to move towards a progressively richer, more conceptual set of themes. Analysis progressed iteratively through initial line-by-line coding and higher-order theme development. We periodically checked themes against codes and extracts to ensure the conceptual analysis remained ideographically grounded in the accounts. While sensitization to the concepts of well-being, resilience and protective processes influenced our research questions and our interrogation of the data in late stages of analysis, we sought to remain open to participants’ nuanced interpretations of how their experiences were adaptive to a sense of subjective well-being. This process resulted in six conceptually coherent themes clustered within a superordinate theme.

We examined our analysis according to criteria for validity and quality in qualitative research (Shaw, 2010; Yardley, 2000). We demonstrated *sensitivity to context* by emphasizing multiple dimensions of participants’ experiences and engaging with the data. We iteratively reflected on our experiences, interpretations, and social context to be sensitive to the effects of our own subjectivities on the analysis (Shaw, 2010). We demonstrated *transparency and coherence* by clearly presenting the evidentiary basis of each theme and its development. We demonstrated *commitment and rigour* through independent coding of five transcripts by a colleague and ongoing discussion between authors. Confluences and discrepancies were discussed to achieve a consistent interpretation. *Impact and importance* were demonstrated by using the findings to inform a subsequent health intervention (de Visser et al., 2015).

## Results

**Figure 1.**
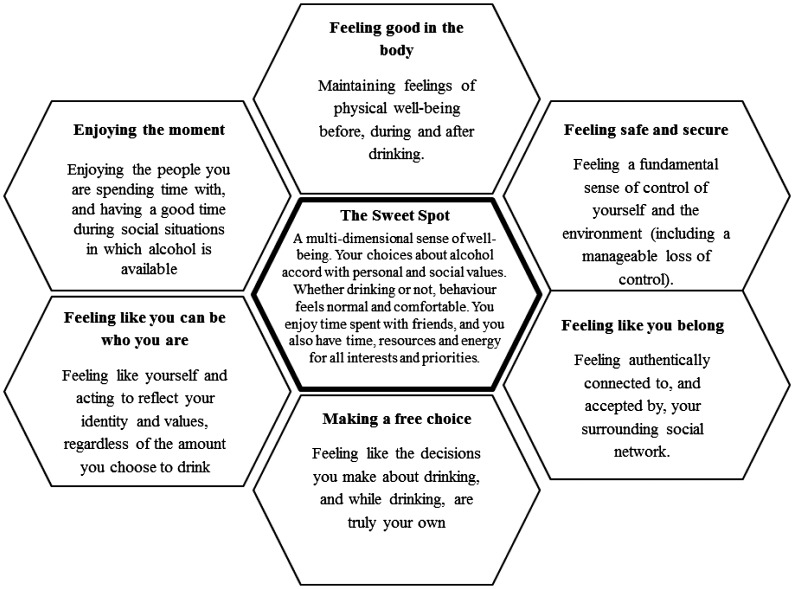
The ‘sweet spot’ cluster and themes.

The analysis identified six themes clustered around a central organising concept of ‘staying in the sweet spot’ (Figure 1). The sweet spot was described as multidimensional, including feelings of enjoyment, control, release, well-being, and a lack of regret while avoiding discomfort, threat or poor mood. The ‘sweet spot’ was a phrase used by John (18+, moderate drinker) when describing why he felt it important ‘not to drink too much’:

It’s important to … find that kind of sweet spot, between, like too sober and too drunk … getting too drunk always tends to end kind of badly … [I] like to get drunk enough … open and friendly and, but, kind of, sensible enough to not do anything too stupid … there’s something like satisfying about, knowing that you’re not drunk enough to do something, but, you’re still having a good time.

John identified some of the key features of the undefined space preceding ‘too drunk’, distilling in a few words feelings which ran through the accounts. This state of mind was identified as ‘the sweet spot’. As suggested within this extract, ‘the sweet spot’ may entail feelings of belonging and security. He feels free to be ‘open and friendly’, allowing a ‘good time’ and still feeling confident in his choices. By not drinking too much, he may pre-empt the option for the night to end ‘badly’. He also identified a form of discomfort (‘too sober’) which suggests that abstention could feel undesirable in certain circumstances.

Gaby (16–17, moderate drinker) identified other key features of the ‘sweet spot’:

[I prefer] having full control of everything and [being] on a high I guess, rather than having that one drink too much and then just kind of sort of coming down … and wanting to go home ‘cos you’re ill.

Feeling good, for Gaby, also involved feelings of security and making free choices, but also touched upon a desire to feel physically well and to enjoy the moment. The language of being on a ‘high’ and ‘coming down’ evokes a sense of threshold. The assurance of knowing she can stay on a ‘high’ is worth refraining from the ‘one drink too much’. Rather than experiencing moderation negatively as a restriction – a mechanism of withholding – moderation is experienced positively as a means of staying in a good place – a mechanism of facilitation.

Angela (18+, moderate drinker) identified the remaining key features of the ‘sweet spot’:

I like letting loose and I like being comfortable, but I don’t mind the being a bit tipsy, that’s kind of fine, but I can’t, I wouldn’t feel comfortable about being drunk.

In this extract, Angela conveyed a strong sense of knowing herself, differentiating between being able to be herself (‘letting loose’), and feeling discomfort. She appears less relaxed in this liminal space than John and Gaby (‘I don’t mind’, ‘kind of fine’) but again identifies moderation as a way to maintain feeling comfortable: in this case, through being herself.

In summary, these young people generally experienced ‘feeling good’ as staying in a ‘sweet spot’ along one or more thematic dimensions: feeling good in the body, feeling like you belong, making a free choice, feeling like you can be who you are, enjoying the moment, and feeling safe and secure. We now describe each in turn.

### Theme 1: Feeling good in the body

Participants expressed a desire to ‘feel good in the body’ before, during and after drinking. Many participants wished to maintain physical well-being by avoiding the anticipated physical consequences of excessive drinking. Heather (16–17, moderate drinker) said:

anything past [tipsy] I sort of get really tired and a bit grumpy, and I just don’t really feel like partying very much … [so I want to stay in] that stage between tipsy and drunk where, where you sort of get really happy, and you’re up for dancing and stuff.

Heather, like many other participants, experienced mild alcohol use pleasurably, but was unwilling to pass through a tipping point, beyond which additional alcohol would lead to unpleasant sensations such as illness, loss of control, vulnerability, dehydration or regretted physical or sexual activities. Enjoyment acted as an incentive to avoid excess. For many participants including Heather, moderate, but not excessive, alcohol use was generally associated with pleasurable sensations such as relaxation, heightened sensation, giddiness, expression and disinhibition. Participants reported that they sometimes had to learn that pleasurable sensations such as relaxation or disinhibition could be achieved without alcohol.

Enjoying the taste of drinks was also an important component of feeling good in the body:

The drinks are usually … horrible but if you’re on a tight budget, you’ll buy one anyway, because you know it’s best to have a little bit of a drink to just kind of calm down a bit, ‘cos there’s always the euphoria of being in a club for the first time you feel like oh I can’t dance … If I’m out with a friend maybe just at a pub, I like to have a beer that actually tastes nice’ – *Gareth, 18*+*, moderate drinker*.

In this extract, Gareth shifted from experiencing drinks as instruments to facilitate well-being, rather than objects of enjoyment in themselves. Prioritizing sensory pleasures led him to moderate his intake, choosing quality over quantity. His discomfort and anticipation in the club appear key to this utilitarian view of alcohol, contrasting with the pub setting which emphasized pleasure and social connection. Some moderate drinkers such as Angela and Gareth found their discomfort in social situations was eased by shifting to consuming smaller amounts of more enjoyable alcoholic drinks or non-alcoholic drinks: ‘I had like one [cocktail] and that was … enough for me to just be like oh, I’m fine, I don’t need anything else’ (Angela, 18+, moderate drinker). Other moderate and former drinkers felt that learning to enjoy the taste of certain drinks, or to drink despite not enjoying the taste, was associated with feelings of youth and inexperience. Learning to drink entailed training themselves to enjoy or tolerate drinks that did not taste pleasurable, compromising feeling good in the body.

In summary, many participants experienced ‘feeling good in the body’ as enjoying feelings of pleasure and exhilaration which they sought to preserve. They also enjoyed the taste of their drinks, whether they contained alcohol or not.

### Theme 2: Feeling like you belong

‘Feeling like you belong’ meant feeling authentically connected and accepted within one’s interpersonal relationships, especially while drinking.

The ability to maintain a friendship through changes in drinking behaviours could signify relationship integrity. In this extract, Mark (18+, non-drinker) reflected on his enduring relationship with a childhood friend who was briefly unhappy when Mark quit drinking:

In the grand scheme of things it’s not that serious … it’s not like our friendship was based on alcohol.

Referencing the time-frame of their friendship, Mark took a wide view of the ‘scheme of things’, instead of narrowing in on what might otherwise have been a pivotal juncture in the friendship. He situated their friendship on the broader, long-lasting ‘grand scheme’ of intimacy, compatibility and endurance. An implicit option is that the friendship *could* have been based on alcohol, as suggested by Iris (18+, moderate drinker): ‘The only reason some people want me in their lives is [to] have a companion to get wasted … that’s not really what friendship should be about’. For Iris, alcohol masked incompatibility. Loss of control during drinking was perceived by many moderate drinkers to reveal a shared vulnerability, making the exhilaration of being ‘wasted’ seem like true friendship. Iris asserts it certainly is not. Mark’s account draws particular attention to participants’ common concern that cutting down drinking may threaten a friendship by introducing the question of what the basis of the friendship actually is. For Mark, maintaining his friendship was associated with emotional authenticity and stronger bonds.

With this threat in mind, refusing drinks tactfully was important to many participants. Returning to Mark: ‘I think my friends … respected when I said no because they knew I [would] get in a bit of a state … [by saying no] you’re kind of a dampener to fun … instead of just saying no and potentially seeming rude it’s sometimes best to … offer a reason’. Without negative judgement Mark suggested that drink refusal might be confused with ‘fun’ refusal. Providing a reason for refusal maintains mutual respect: his friends value his well-being and he values their perspectives. Avoiding being in a ‘state’ may facilitate belonging, since sobriety will prevent him being too incapacitated to socialise.

Being ‘seen’ as belonging was important for actually *feeling* like one belonged. Kathryn (18+, non-drinker) relates:

a lot of my friends [at uni] had known people who hadn’t drunk, whereas [growing up] I was like a complete anomaly … I could’ve been an alien.

Kathryn, a lifelong non-drinker, described her feelings of alienation during her adolescence quite vividly, placing her as coming from a different planet. She was an ‘anomaly’, but this is something more than simply being outnumbered. Being an ‘alien’ may involve not only having different social practices and values which erect barriers to connection, but, strikingly, implies a ‘seen’ physical difference. This seeing may work both ways, with her viewing her peers from a distance. Elsewhere, Kathryn related finding an accepting friendship group who ranged from moderate drinkers to abstainers. Knowing other non-drinkers and having peers who know other non-drinkers made her teetotal status less salient. Drawing attention to differences with peers was an uncomfortable way of being ‘seen’. It could be the catalyst for feelings of isolation, as shown by Vinay (18+, non-drinker): ‘That’s when I felt left out, seeing how many people going to enjoy themselves and me not being able to’. Feelings of connectedness were maintained by Vinay through engaging in activities where alcohol would not be present, and by others as frequenting clubs and pubs while sticking to soft drinks.

In summary, belonging involved feeling that friendships were founded on authenticity. Alcohol was adaptively experienced as demonstrating relationship strength once the social glue of drinking was stripped away. It also facilitated artificial closeness. Being seen as different could involve separation and isolation, particularly for non-drinkers. Participants often valued an accepting peer group with diverse consumption levels.

### Theme 3: Feeling like you can be who you are

‘Feeling like you can be who you are’ meant feeling like yourself before, during and after drinking. Corinne’s account verbalized common concerns over feeling authentic during instances of social drinking:

My friends say they wouldn’t want to meet the drunk version of themselves. I don’t like the idea of there being a part of me that I wouldn’t want to know fully.

Here, Corinne (18+, non-drinker) differentiated between a view of the self as comprising alternative ‘versions’, and a coherent view of the self as a core containing many ‘parts’. She believed that her friends perceive a ‘drunk version’ of themselves as a mask, an inauthentic and fragmented representation of who they really are, creating a distance between this ‘drunk version’ and the implied ‘real self’. By contrast, a ‘drunk’ Corinne would be ‘a part of me’. Self-knowledge is a revelatory process of developing relationships: parts of the self are like friends she can get to know, but they still belong to a whole, retaining a sense of closeness. Being who you are involved feeling like you *know* who you are, and feeling that sense of self resonate through social drinking situations even when exploring new relationships, identities and activities. Alcohol was seen to introduce a fragmentation which ruptures the true nature of the self as whole. In other participants’ accounts this experience was realized through a felt separation between ‘real’ choices when sober or tipsy and choices made ‘only because I was drunk’. Beyond the ‘sweet spot’, participants felt inauthentic or lost.

Recognizing ‘who you are’ required understanding one’s personal values, as Ting (18+, moderate drinker) explained:

Everyone has their own … values, like own ideas and own perception of themselves, like, what to do and what not to do … I do what makes me feel comfortable … instead of like, doing the opposite which makes me feel uncomfortable

For Ting, a strong understanding of her values and a reflective self-awareness reliably guided moderation in drinking. ‘Everyone’ has these resources, but must choose whether to explore and engage with them. In other accounts, self-beliefs incorporated the opinions of friends, community leaders, and family, with pride in accompanying social roles (‘I still want to be a role model’, Gareth, 18+, moderate drinker).

An important part of ‘feeling like you can be who you are’ is feeling that you do not need alcohol to be yourself. Alcohol could nonetheless be experienced as strategically valuable, as shown in this extract from Lucy’s account (16–17, moderate drinker):

when you’re … a bit tipsy, and you feel it’s a bit easier to speak to people … that kind of inhibition was like lowered a bit … it’s just like if this is gonna work I will do it

This extract illustrates a common view in these moderate drinkers’ accounts: that alcohol might enable the courage and sociability needed to feel like yourself. In Lucy’s case, this meant feeling more able to express herself in front of colleagues while socialising. Alcohol could therefore be experienced positively, if not un-problematically, as facilitating self-expression. However, beyond the ‘sweet spot’ could lie a jarring disjunction between self-beliefs and the social presentation of the self – feeling like the ‘drunk version of themselves’ (Corinne) did not match their ‘perception of themselves’ (Ting). This negative feeling of disjunction sometimes prompted self-insight: ‘if I’m not enjoying it … that should tell me something’ (Iris, 18+, moderate drinker). Iris realized that her drinking behaviours were not facilitating her well-being. As in Corinne’s extract, parts of the self were experienced in empathic conversation, ‘telling’ her what she really felt.

In summary, ‘feeling like you can be who you are’ involved feeling a sense of authenticity about the self which carries through relationships, activities and interests. Limited use of alcohol could align with authenticity, but self-knowledge helped participants recognize boundaries beyond which behaviour jarred with who they believed themselves to be.

### Theme 4: Making a free choice

‘Making a free choice’ meant feeling like the decisions made about drinking, and while drinking, were truly one’s own. Making a free choice was also experienced as feeling proud about making drinking choices which reflect one’s personality, values and priorities.

Drinking more than intended could mean feeling like the choice to drink *more* was not really free, as demonstrated by Gaby (16–17, moderate drinker):

Me and my friends literally have no idea what happened ‘cause all of us obviously went out with the same intentions … to stay sober (laughs) and the opposite happening.

As suggested by Gaby’s laughter, a limited loss of control was part of the thrill of drinking. It also entailed risks of getting carried away in the moment. For Gaby and others, intentions set when sober might be overpowered by the physical effects of alcohol, the seduction of feeling even better by drinking more, or social encouragement. For some, drinking involved a voluntary, limited loss of control, with feelings of exhilaration, escape, and unpredictability. However, choosing to lose control could entail regret or victim-shaming when negative consequences were perceived. Many identified this as incentive to limit their drinking.

Participants distinguished between engaging with norms about drinking and experiencing autonomy in social situations. Returning to Gaby’s account:

When people talk about peer pressure to drink I’m just like ‘doesn’t exist’. I’ve never felt any pressure to, I do it because I’ve chosen to, not because someone’s forced me to … [‘peer pressure’] sounds like people are just like ‘Drink drink drink’ … I’ve never had someone be like that to me, or … it was only jokingly.

Gaby suggested that her actions were not directly affected by social pressures in specific interactions. She, like nearly all participants, was generally resolute that her choices were ultimately her own. Instead, she experienced expectations for cultural practices, such as being ‘jokingly’ egged on. Participants reported experiencing expectations to drink through socialising at pubs and parties, purchasing rounds or playing drinking games, fixed ideas of a ‘good night out’ and wanting to experience excess in order to develop personal boundaries.

Many accounts also referenced cultural norms of drinking as an important part of emerging young adult socializing, associated with freedom from parents or authority figures. However, this could contribute to a ‘teenage drama-y atmosphere’ in which peers were overly interested in one’s personal choices about alcohol: ‘at first some people got pissed off, but then … everyone was like well she can do what she likes’ (Lucy, 18+, moderate drinker). Choosing to drink can, in such situations, signify autonomy. For Lucy, and particularly amongst abstainers, choosing *not* to drink was experienced as freethinking individuality. Social acceptance and admiration were not required, but were welcomed.

Participants experienced ‘making a free choice’ as feeling that their choices were truly their own. Choices were contextualized within positively-experienced desires to cede control in thrilling situations, and more limiting norms.

### Theme 5: Enjoying the moment

‘Enjoying the moment’ was perhaps the most constant dimension of well-being across participant accounts, experienced as joy or contentment in activities and relationships, regardless of the amount of drinking.

At its most basic, enjoying the moment involved, simply, *fun*. Gaby bluntly illustrated the contrasting case: ‘there’s no need to get yourself into that state, it’s not enjoyable, you’re gonna get taken home, miss what you paid £200 for’ (Gaby, 16–17, moderate drinker). If drinking should be fun, then moderating intake in order to enable enjoyment was perceived as desirable, if sometimes hard to achieve. Like Gaby, participants across ages and consumption levels perceived excess as a waste of time and resources, and many reflected on whether or not pubs and clubs were enjoyable places.

More deeply, ‘enjoying the moment’ involved feeling that the experience of fun was not dependent on alcohol, as indicated by Corrine’s extract:

I’ve enjoyed being with my friends and I’ve enjoyed like dancing and spending time with them … when they drink they become more fun in some ways … and I’m happy for those people. But I don’t feel like I need [it].

For Corinne (18+, non-drinker), ‘feeling good’ meant feeling connected, enjoying the situation, and enjoying her friends’ company even when they had consumed alcohol. The underlying work involved in building a good moment, in the immediate sensory and social experience, appeared in her sense of security in interpersonal bonds and authenticity in self-expression. As a non-drinker who proudly socialized primarily with drinkers, Corinne’s extract draws attention to the centrality of participants’ experiences of shared enjoyment with friends to the ‘sweet spot’. Her tone demonstrates a loving and non-judgemental attitude towards her friends, while directly acknowledging her own reservations about drinking.

Corrine’s extract also hints at the work of building a good moment, exemplified in this extract from Angela (18+, moderate drinker):

I know in my heart of hearts actually I’ve got it going good because I do my art work, I have my friendship groups … I can enjoy being with them, with or without alcohol.

For Angela, ‘going good’ was experienced as having other meaningful interests, preferences and priorities, that facilitated personal expression, creativity and social connectedness. Having friends with interests beside alcohol was important for Angela, as it was for a number of participants. Being able to identify ‘going good’ involved an awareness of what she found fulfilling (in her ‘heart of hearts’). Self-knowledge might direct a person towards other ways of ‘enjoying the moment’, so that alcohol becomes incidental. Conversely, refusing alcohol may also bring self-knowledge: particularly within accounts of young men non-drinkers, this choice was experienced as opening up new social worlds through activities such as fitness and music. Saying ‘no’ to excessive drinking could mean saying ‘yes’ to other experiences.

Enjoying the moment, therefore, involved an immediate sense of fun and having time and resources to pursue valued interests unrelated to drinking. Social life and self were not primarily defined by drinking practices. In this way, refusing alcohol was experienced less as *abstaining from* an experience of social drunkenness, and more as *facilitating* enjoyment.

### Theme 6: Feeling safe and secure

‘Feeling safe and secure’ meant feeling a fundamental sense of control about oneself and one’s environment. This might involve exploring, discovering and challenging boundaries within a safe environment, as demonstrated in this extract from Joseph’s account:

My friends are there so that if anything does happen … I just feel very comfortable, I feel able to try things really … [Sometimes] if I get drunk [other] people are going to give me a hard time, and probably make me do something silly and embarrass me, and those people are probably going to look after me … I don’t want to be in either of those places so I’m not going to drink.

Joseph (16–17, moderate drinker) felt able to ‘try things’ within the realm of normative risks, bolstered by reassurance that his friends were there to help. He expressed pride in refusing alcohol when drinking might exploit his friends’ good will or introduce uncomfortable levels of social risk. Joseph’s extract illustrates how feeling safe and secure is not restricted to concerns about sexual predation, criminal or antisocial activity, and physical safety, although these concerns underlie many participants’ experiences. Participants also expressed concerns about negative *social and emotional experiences* (e.g. regretted sexual activity, insults, or disclosures). ‘Feeling safe and secure’ therefore extended to emotional and social security. As shown here, security involved feeling connected to reliable caretakers such as friends, parents, siblings, and medical personnel. Avoiding risk entirely was not desirable to Joseph: he valued feeling able to ‘try things’. Attraction to managed risk ran through the accounts of many moderate drinkers: ‘feeling safe and secure’ could involve a temporary, exploratory and experimental loss of control, with no lasting emotional, instrumental or physical effects upon the self, others or valued priorities. Advance planning could facilitate this sense of security, such as through discussions with friends about how to party safely, arranging lifts home, or testing out a club with a more experienced friend.

Feeling safe and secure could also mean feeling available to *provide* support, as Joseph suggested later: ‘my friends very much respect me for thinking that [supportive] way’. It was important, however, that this contribution be valued and reciprocal: ‘my role on a night out was always to be like the primary carer, and people’s friends, parents would be happy with that ... because they knew their kids would be ok, and I was like, this isn’t really fair’ (Kathryn, 18+, non-drinker). Kathryn resented this exploitation of her caretaking role. Whereas Joseph experienced inclusion within the friendship group through his support provision, Kathryn experienced division from her peers, reducing her presence from friend to a ‘carer’ ‘role’ in substitute for parental supervision. As with other themes, feelings of authenticity and connection can be identified here as guideposts through which safety behaviours may be experienced as enabling well-being, instead of placing restrictions.

‘Feeling safe and secure’ involved negotiating between challenge and thrill-seeking on one hand, and comfort and stability on the other. Safely losing control could feel exhilarating. Feeling able to receive and provide support through valued relationships was important to sustaining a feeling of enjoyable, and safe, social interaction.

## Discussion

A resilience-based analysis identified a ‘sweet spot’ of multidimensional subjective well-being characterized by feelings of enjoyment, control, release, and a lack of regret with respect to low-risk drinking and abstention. The sense of balancing enjoyment with restraint to continue to experience well-being was captured in the thematic cluster of the ‘sweet spot’ and its six composite dimensions. *Feeling good in the body* entailed feeling good physically before, during and after drinking. *Feeling like you belong* involved feeling authentically connected to and accepted by one’s significant others. *Making a free choice* meant feeling like the decisions made about drinking, and while drinking, are truly one’s own. *Feeling like you can be who you are* entailed feeling that the self expressed when drinking is really ‘you’. *Enjoying the moment* was experienced as having a good time regardless of the amount consumed. Finally, *feeling safe and secure* meant feeling a sense of control about the self and the environment. These dimensions varied in significance and expression across individuals but were generally present to some degree in all accounts.

### Staying in the ‘sweet spot’: Resilience in a drinking culture

Within the frame of meaning-making offered by our analytical method, we suggest that the ‘sweet spot’ is a coherent conceptual rendering of subjective alcohol resilience among young people in a drinking culture. Whether this corresponds to observable behaviour and measures of psychological and physical health is a matter for future research. However, among participants who were below the threshold for high-risk alcohol consumption, the ‘sweet spot’ captured lived experience of subjective well-being in social situations where drinking was an option. It incorporates meaning-making and perceived growth in the face of challenging encounters, relationships and spaces, situated against a cultural context of alcohol-based risk. Yet ‘feeling good’ is not a passive state: participants’ experiences of *maintaining* ‘the sweet spot’ capture a sense of proactivity and agency. Ultimately, alcohol resilience is more than alcohol refusal, although it is that, too: it is actively choosing to stay in one’s ‘sweet spot’.

### Exploring protective processes

The findings suggest the utility of developing experienced-based definitions of optimal drinking experiences, capturing resilience in the face of culturally-situated alcohol risk. The ‘sweet spot’ may be a useful guiding concept, providing a positive experience to aim for and reassurances of feasibility that support behaviour change (Abraham et al., 2007). A coherent subjective definition of healthy and adaptive experiences may help young people to recognize and develop skills and resources that facilitate resilience within cultures of normative alcohol use. Analysis highlighted experiential thematic dimensions that were meaningful for ‘feeling good’ in the face of cultural alcohol-related risk. The complexity of participants’ lived experiences reflect different priorities, values and strengths. Critically, complementary or competing values may characterise lived experience. For example, an adolescent who drinks moderately when socialising is not necessarily poor at making choices: he/she may prioritise feelings of belonging, which are valuable to positive adaptation (Hart et al., 2007). We now explore how our analysis points towards protective processes in three domains: adaptive social influences, self-based strengths, and the ability to balance a threshold of well-being.

#### Adaptive social influences

The analysis revealed that, contrary to much prior research, peer influences may be experienced as adaptive in the realm of alcohol use. The risks of peer influence are well-documented: alcohol consumption is associated with a peer-oriented lifestyle and peers’ consumption (de Visser et al., 2013; Kokkevi et al., 2007). Yet our findings suggest that friendships may also enable belonging, security and freedom of choice. Authentic friendships were those that thrived despite changing drinking patterns or differences in friends’ alcohol use. Excessive drinking was viewed as interfering with meaningful communication whereas moderate use could facilitate belonging. Interviewees described how friends provided reassurance, made practical arrangements, and supported each other through challenging situations. Participants valued having a varied, accepting social network. By contrast, participants experienced norms as incentives and social practices encouraging excess. Non-drinkers and moderate drinkers have previously identified the simultaneous importance of developing a social life independent from alcohol and the ability to integrate into drinking situations (Herring et al., 2013). This paradox of integration and alienation was reflected here. Findings indicate that successful differentiation – drink refusal in a drinking culture – facilitated social integration in a way that minimized the social significance of alcohol, respected individuals’ choices and enabled connection.

#### Self-based strengths

Many frameworks of youth resilience (e.g. Hart et al., 2007) emphasize a sense of self, effective coping, and the ability to learn from challenges in life, which are demonstrated by an individual even as they involve the support of significant others and negotiation with the environment. Participants demonstrated a number of self-based strengths. ‘Feeling like you can be who you are’ was tied into having a strong sense of self-concept, experienced as a core self. ‘Core self’ appears in other literature – for example, to describe individuals’ appraisals of themselves as worthy, effective and capable (Judge, Erez, Bono, & Thoresen, 2003) and, in a usage more relevant for this study, as a focus of efforts to foster young people’s talents, self-knowledge, hope, and self-responsibility (e.g. Hart et al., 2007). As used here to interpret our participants’ meaning-making, ‘core self’ denotes a phenomenology of the self as a coherent and authentic whole, which may nonetheless be composed of many parts. Participants spoke with self-compassion and intimacy about their core self. This feeling of core self may be contrasted with a fragmented sense of self in which different parts felt unknown, disparate or inauthentic during drinking situations – a subjective indicator of having moved beyond the ‘sweet spot’. Self-efficacy was suggested as participants sought to feel free in their choices while negotiating norms, physical exhilaration, and concerns with autonomy and social acceptance. Fragmentation was experienced with discomfort, suggesting implications for young people’s self-esteem. Self-insight could occur as participants wrestled with feelings that drinking could alter, obscure or strip bare the self, while paradoxically believing drinking could facilitate self-expression. Within the ‘sweet spot’, alcohol has an incidental relationship with the self; beyond the sweet spot, its role becomes instrumental. This distinction resonates with previously-identified differentiation between experiences of drinking to relax and drinking to cope (Bernstein et al., 2011).

#### Balancing a threshold of well-being

Analysis highlighted a unique challenge for moderate drinkers: they must first *start* drinking, then *stop*. This dance between engagement in and disengagement from a behaviour is more broadly reflected in participants’ experiences of balancing a threshold of well-being. We suggest that one novel component of alcohol resilience is adeptness with balancing competing desires and coming to a resolution which lies within the ‘sweet spot’. Participants negotiated desires to feel safe and take risks; to feel present and alter the senses; to feel exhilarated and avoid sickness; to conserve and push boundaries. This sense of threshold was particularly evident in ‘feeling good in the body’ and ‘feeling safe and secure’. It also involved feeling present to experience well-being, even amid experimentation. Important facilitators of this experience were practical planning in relation to safety, using social support and self-knowledge to practice a boundaried loss of control and using embodied experience to monitor well-being. The thresholds of well-being may be rendered visually as the boundaries of the hexagons in Figure 1: while exploration into risk-taking, sensory alteration, and social experimentation may feel good, there is a subjective threshold beyond which lie physical discomfort, inauthenticity, alienation, restriction, danger and lack of enjoyment. This experience may be intuitively recognisable, but further studies might develop this construct and its behavioural correlates more thoroughly.

### Implications for policy and intervention development

The resilience-based approach used here invites conversation about strengths-based positive approaches to preventing problems of alcohol use that incorporate young people’s subjective perspectives (Cheon, 2008). A framework of positive adaptation may help construct realistic recommendations for feasible behaviour patterns given the low to moderate efficacy of many behavioural interventions targeting young people’s alcohol use. Even brief interventions can be effective, particularly in schools (Carney, Myers, Louw, & Okwundu, 2014; Healey et al., 2014). Engagement with lived experience may help to avoid patronizing young people, develop realistic recommendations for handling common situations and build on adaptive significant relationships. Young people’s drinking choices are experienced through values, embodiment, social connections, and perceptions of context-specific drinking practices. This multidimensionality lends itself to complex health interventions using a variety of resilience-building techniques (de Visser et al., 2015). Practitioners might use a range of behaviour change techniques (Abraham & Michie, 2008) to support development of protective mechanisms and their application along the six thematic dimensions in ways that allow individuals to prioritize their personal relevance.

Our findings suggest that interventions focusing on developing social integration, social support, sense of core self, self-efficacy, self-monitoring and planning may be particularly effective in developing resilience in the face of cultural alcohol risk. In the domain of developing *adaptive social influences,* psychosocial interventions may aim to build psychosocial skills, strengthen accuracy of social drinking norms and shift perceptions among friendship groups regarding drinking quantities, frequency and social facilitation (Foxcroft & Tsertsvadze, 2012; Moreira, Smith, & Foxcroft, 2009). These types of interventions can reduce excessive drunkenness and binge drinking (Fletcher, Bonell, & Hargreaves, 2008; Foxcroft & Tsertsvadze, 2012; Moreira et al., 2009). We suggest working with desires for belonging, authenticity in relationships, and integration, and acknowledging fears around alienation and the fragility of relationships. Young people could be supported to learn strategically how to refuse alcohol, assess relationship quality, cope with changes in relationships and develop a varied, non-judgmental social group. Mutual planning and emotional reassurance should be facilitated. Communities must offer spaces for socialising where alcohol use would be incidental or irrelevant and in which meaningful long-term social relationships can flourish (Roberts et al., 2012).

Interventions might focus on *self*-*based strengths* while acknowledging the social nature of alcohol use to calibrate feasibility and explore anticipated barriers to change. The importance of ‘making a free choice’ supports previous indications of the utility of enhancing moderate drinking self-efficacy (Atwell, Abraham, & Duka, 2011). Other interventions have effectively targeted self-efficacy, drink refusal skills and vocational skills (Fang & Schinke, 2013; Griffin, Holliday, Frazier, & Braithwaite, 2009; Johnson et al., 1998). Incorporating coping support and social experience may bolster the efficacy of such interventions. Crucially, our findings highlight self-knowledge as a foundation for well-being. Young people could be supported to develop and appreciate their core self, even as they explore their evolving identities, and to recognize when the choices they make feel in accordance with that core self. Such developments may extend to making positive behavioural choices in other health-related domains.

Finally, we suggest exploring interventions focused on *balancing a threshold of well*-*being.* This construct resonates with previous research that has identified adaptive functions of moderate social drinking experiences such as social facilitation, altering mood, stress relief, or providing temporary escape from difficulties (Bernstein et al., 2011; de Visser & Smith, 2007; de Visser et al., 2013; Pavis, Cunningham-Burley, & Amos, 1997). We propose that successful interventions should engage with young people’s positive and negative beliefs about social drinking, normative risk-taking, and play with identity and sensory experience to discuss how to maintain safety, security, authenticity, and physical and psychological well-being. Young people wishing to drink moderately should be supported to manage the difficult position of starting to drink, and then stopping.

### Limitations and future research directions

We acknowledge limitations to this research, which in turn suggest directions for future research exploring the concept of the ‘sweet spot’ and clarifying its relationship to drinking patterns and other health behaviours. First, this study aimed to explore subjective experiences. We therefore did not assess individuals’ alcohol consumption beyond using prior reports of alcohol intake to inform our sampling by categorizing participants as non-drinkers or moderate drinkers. Using objective consumption criteria to direct investigation of a subjective experience is a valuable use of a pragmatic mixed-methods approach (de Visser et al., 2015). The ‘sweet spot’ was an emergent concept developed through rigorous analysis, not an a priori categorical tool. There is necessarily a tension between the use of objective measures, and the nuances and complexities of lived experience which elude even the most robust psychometric measure. The next step is therefore to explore links between the ‘sweet spot’ and behavioural measures of alcohol consumption, psychological well-being and physical health. For example, it would be useful to know if there are unit-based correlates of sensations and behaviours related to the ‘sweet spot’, whether moderate drinkers who experience the ‘sweet spot’ may nonetheless be drinking to a harmful level, whether heavy drinkers experience the ‘sweet spot’ at any time during their drinking, and the circumstances in which even resilient young people engage in harmful drinking behaviours. Longitudinal qualitative and quantitative research may be used to determine the long-term psychological and medical consequences of drinking to within, or beyond, one’s ‘sweet spot’.

Second, our findings may be argued to promote alcohol use by positioning moderate alcohol use within the realm of healthy behaviour patterns. We caution against this interpretation. In the absence of changes to legislation, cultural alcohol risk may never disappear entirely. Positive adaptation is a continual process of experiencing psychological change as one encounters and responds to challenges. It may be therefore be useful to complement abstention approaches with a harm-minimisation approach aimed at the wider population of general alcohol drinkers (Danielsson et al., 2011): identifying a range of healthy behaviour patterns and facilitating the development of skills to attain these. The approach presented here is designed to complement, but not replace, existing effective approaches such as market regulation, advertising bans and family-based interventions (Anderson, Chisholm, & Fuhr, 2009; Foxcroft & Tsertsvadze, 2012). Researchers and practitioners should identify young people at high risk for alcohol-related harm and refer them to appropriate interventions, a process which is currently reliant on emergency departments (Healey et al., 2014). We suggest that young people who feel they regularly go beyond their Sweet Spot are likely to engage in maladaptive behaviours which may place them at high risk of alcohol-related harm. Open, non-stigmatizing discussions of adaptive and maladaptive behaviours may be a way of identifying young people who are likely to be at high risk.

Finally, it is unclear whether the experience of the ‘sweet spot’ is specific to alcohol use. Research using other age groups, cultural contexts or methodologies might yield different findings. It is important to continue to explore the potential adaptive benefits of friendships and peer groups. Researchers might explore whether young people who are skilled at staying within their ‘sweet spot’ better manage other health risks in which peers and cultural norms influence individual behaviour, such as smoking, substance use and sexual risk-taking (Avry, Duncan, Duncan, & Hops, 1999; Tyas & Pederson, 1998). Researchers might examine how different dimensions of the ‘sweet spot’ interact: for example, by empowering young people to make choices which reflect one’s core self. Transferability of adaptive processes in the development and maintenance of alcohol resilience would be encouraging for designers of health interventions seeking to achieve maximal and diffuse positive effects.

## Conclusions

We suggest that the construct of the ‘sweet spot’ is a coherent conceptual rendering of subjective alcohol resilience among young people in a drinking culture. Our findings highlight the importance of adaptive social relationships, self-based strengths and balancing a threshold of well-being across six identified dimensions of optimal drinking experience. Researchers and practitioners are encouraged to continue engaging with young people’s lived experience in order to develop effective behavioural interventions and community-based policies to improve individuals’ psychological well-being and minimise alcohol-related harm.

## References

[CIT0001] Abraham C., Michie S. (2008). A taxonomy of behaviour change techniques used in interventions. *Health Psychology*.

[CIT0002] Abraham C., Southby L., Quandte S., Krahé B., van der Sluijs W. (2007). What’s in a leaflet? Identifying research-based persuasive messages in European alcohol-education leaflets. *Psychology & Health*.

[CIT0003] Anderson P., Chisholm D., Fuhr D. (2009). Effectiveness and cost-effectiveness of policies and programmes to reduce the harm caused by alcohol. *Lancet*.

[CIT0004] Atwell K., Abraham C., Duka T. (2011). A parsimonious, integrative model of key psychological correlates of UK university students' alcohol consumption. *Alcohol and Alcoholism*.

[CIT0005] Avry D. V., Duncan T. E., Duncan S. C., Hops H. (1999). Adolescent problem behaviour: The influence of parents and peers. *Behaviour Research and Therapy*.

[CIT0006] Babor T. F., Higgins-Biddle J. C., Saunders J. B., Monteiro M. G. (2001). *The alcohol use disorders identification test: Guidelines for use in primary care*.

[CIT0007] Balogun O., Koyanagi A., Stickley A., Gilmore S., Shibuya K. (2014). Alcohol consumption and psychological distress in adolescents: A multi-country study. *Journal of Adolescent Health*.

[CIT0008] Bernstein J., Graczyk A., Lawrence D., Bernstein E., Strunin L. (2011). Determinants of drinking trajectories among minority youth and young adults: The interaction of risk and resilience. *Youth and Society*.

[CIT0009] Carney T., Myers B. J., Louw J., Okwundu C. I. (2014). Brief school-based interventions and behavioural outcomes for substance-using adolescents. *Cochrane Database of Systematic Reviews*.

[CIT0010] Cheon J. W. (2008). Best practices in community-based prevention for youth substance reduction: Towards strengths-based positive development policy. *Journal of Community Psychology*.

[CIT0011] Conroy D., de Visser R. O. (2013). Being a non-drinking student: An interpretative phenomenological analysis. *Psychology and Health*.

[CIT0012] Danielsson A.-K., Wennberg P., Hibell B., Romelsjö A. (2011). Alcohol use, heavy episodic drinking and subsequent problems among adolescents in 23 European countries: Does the prevention paradox apply?. *Addiction*.

[CIT0013] Department of Health (2009). Guidance on the consumption of alcohol by children and young people.

[CIT0014] Department of Health (2013). Reducing harmful drinking: Policy.

[CIT0015] de Visser R. O., Graber R., Hart A., Abraham C., Memon A., Watten P., Scanlon T. (2015). Using qualitative methods within a mixed-methods approach to developing and evaluating interventions to address harmful alcohol use among young people. *Health Psychology*.

[CIT0016] de Visser R. O., Hart A., Abraham C., Graber R., Scanlon T., Memon A. (2014). How alike are young non-drinkers, former-drinkers, low-risk drinkers, and hazardous drinkers?. *Addictive Behaviors*.

[CIT0017] de Visser R. O., Smith J. A. (2007). Alcohol consumption and masculine identity among young men. *Psychology & Health*.

[CIT0018] de Visser R. O., Wheeler Z., Abraham S. C. S., Smith J. A. (2013). Drinking is our modern way of bonding: Young people’s beliefs about interventions to encourage moderate drinking. *Psychology & Health*.

[CIT0019] Dodd L., Al-Nakeeb Y., Nevill A., Forshaw M. (2010). Lifestyle risk factors of students: A cluster analytic approach. *Preventive Medicine*.

[CIT0020] Fang L., Schinke S. P. (2013). Two-year outcomes of a randomized, family-based substance use prevention trial for Asian American adolescent girls. *Psychology of Addictive Behaviors*.

[CIT0021] Fletcher A., Bonell C., Hargreaves J. (2008). School effects on young people’s drug use: A systematic review of intervention and observational studies. *Journal of Adolescent Health*.

[CIT0022] Foxcroft D. R., Tsertsvadze A. (2012). Universal alcohol misuse prevention programmes for children and adolescents: Cochrane systematic reviews. *Perspectives in Public Health*.

[CIT0023] Frederiksen N., Bakke S., Dalum P. (2012). No alcohol, no party: An explorative study of young Danish moderate drinkers. *Scandinavian Journal of Public Health*.

[CIT0024] Griffin J. P., Holliday R. C., Frazier E., Braithwaite R. L. (2009). The BRAVE (Building Resiliency and Vocational Excellence) program: Evaluation findings for a career-oriented substance abuse and violence preventive intervention. *Journal of Health Care for the Poor and Underserved*.

[CIT0025] Hart A., Blincow D., Thomas H. (2007). *Resilient therapy: Working with children and families*.

[CIT0026] Healey C., Rahman A., Faizal M., Kinderman P. (2014). Underage drinking in the UK: Changing trends, impact and interventions: A rapid evidence synthesis. *International Journal of Drug Policy*.

[CIT0027] Herring R., Bayley M., Hurcombe R. (2013). ‘But no one told me it’s okay not to drink’: A qualitative study of young people who drink little or no alcohol. *Journal of Substance Use*.

[CIT0028] Johnson K., Bryant D. D., Collins D. A., Noe T. D., Strader T. N., Berbaum M. (1998). Preventing and reducing alcohol and other drug use among high-risk youths by increasing family resilience. *Social Work*.

[CIT0029] Judge T., Erez A., Bono J., Thoresen C. (2003). The core self-evaluations scale: Development of a measure. *Personnel Psychology*.

[CIT0030] Kloep M., Hendry L. B., Ingebrigtsen J. E., Glendinning A., Espnes G. A. (2001). Young people in ‘drinking’ societies? Norwegian, Scottish and Swedish adolescents’ perceptions of alcohol use. *Health Education Research*.

[CIT0031] Kokkevi A., Arapaki A., Richardson C., Florescu S., Kuzman M., Stergar E. (2007). Further investigation of psychological and environmental correlates of substance use in adolescence in six European countries. *Drug and Alcohol Dependence*.

[CIT0032] Kuntsche E., Knibbe R., Gmel G., Engels R. (2005). Why do young people drink? A review of drinking motives. *Clinical Psychology Review*.

[CIT0033] Kuntsche E., Rehm J., Gmel G. (2004). Characteristics of binge drinkers in Europe. *Social Science and Medicine*.

[CIT0034] Luthar S. S., Cicchetti D., Becker B. (2000). The construct of resilience: A critical evaluation and guidelines for future work. *Child Development*.

[CIT0035] Moreira M. T., Smith L. A., Foxcroft D. (2009). Social norms interventions to reduce alcohol misuse in university or college students (review). *Cochrane Database of Systematic Reviews*.

[CIT0036] Nolen-Hoeksema S., Wisco B. E., Lyubomirsky S. (2008). Rethinking rumination. *Perspectives on Psychological Science*.

[CIT0037] Pavis S., Cunningham-Burley S., Amos A. (1997). Alcohol consumption and young people: Exploring meaning and social context. *Health Education Research*.

[CIT0038] Philips-Howard P., Bellis M., Briant L., Jones H., Downing J., Kelly I., Bird T., Cook P. (2010). Wellbeing, alcohol use and sexual activity in young teenagers: Findings from a cross-sectional survey in school children in North West England. *Substance Abuse Treatment, Prevention and Policy*.

[CIT0039] Rehm J., Mathers C., Popova S., Thavorncharoensap M., Teerawattananon Y., Patra J. (2009). Global burden of disease and injury and economic cost attributable to alcohol use and alcohol-use disorders. *The Lancet*.

[CIT0040] Roberts M., Townshend T., Pappalepore I., Eldridge A. (2012). *Local variations in youth drinking cultures*.

[CIT0041] Rutter M., Rolf J., Masten A. S., Cicchetti D., Neuchterlein K., Weintraub S. (1990). Psychosocial resilience and protective mechanisms. *Risk and protective factors in the development of psychopathology*.

[CIT0042] Shaw R. (2010). Embedding reflexivity within experiential qualitative psychology. *Qualitative Research in Psychology*.

[CIT0043] Smith J. A., Flowers P., Larkin M. (2009). *Interpretative phenomenological analysis: Theory, method and research*.

[CIT0044] Tyas S. L., Pederson L. L. (1998). Psychosocial factors related to adolescent smoking: A critical review of the literature. *Tobacco Control*.

[CIT0045] Yardley L. (2000). Dilemmas in qualitative health research. *Psychology and Health*.

